# Inequalities in utilization and provision of dental services: a scoping review

**DOI:** 10.1186/s13643-021-01779-2

**Published:** 2021-08-10

**Authors:** Arash Ghanbarzadegan, Peivand Bastani, Liana Luzzi, David Brennan

**Affiliations:** 1 Australian Research Centre for Population Oral Health, Adelaide Dental School, The University of Adelaide, Adelaide, SA 5000 Australia; 2 Health Human Resources Research Centre, School of Health Management and Medical Informatics, Shiraz University of Medical Sciences, Shiraz, Iran

**Keywords:** Disparities, Oral health, Developed countries

## Abstract

**Background:**

There are many determinants that can affect inequality in oral and dental health. This study is aimed to explore the main determinants of inequality in both utilization and provision of dental services in Organization for Economic Co-operation and Development (OECD) countries.

**Methods:**

Four databases including PubMed, ISI WOS, Scopus, and ProQuest were searched up to 8 Aug 2020, applying the relevant keywords. Thematic analysis was used for synthesizing and extracting data. Trend analysis was applied to determine the trends of the inequality determinants.

**Results:**

Thematic analysis led to 6 main themes, 13 sub-themes, and 53 sub-sub-themes. The main themes represent the main inequality determinants for both utilization and provision of dental services. The streamgraph illustrated that fewer studies have been conducted on social and cultural determinants, and for almost all determinants the trend of published articles has been increasing since 2007, with the exception of health policies.

**Conclusions:**

Inequality in the utilization and provision of dental services is addressed by various factors including individual, social, cultural and economic determinants, health policies, and availability of services. The first four determinants are related to utilization and the last two are related to the provision of services. All these aspects must be considered to reduce inequality in dental services.

**Supplementary Information:**

The online version contains supplementary material available at 10.1186/s13643-021-01779-2.

## Background

There are multiple determinants which can affect health status. For instance, it has been reported that people of a lower socioeconomic level experience more burden from morbidity and mortality of diseases compared with other groups [1, 2]. Oral health is also believed to be highly associated with socioeconomic status [3]. Dental diseases are among the most prominent public health issues, due to their high universal prevalence and direct effect on quality of life [4].

Inequality in oral health has drawn the attention of worldwide stakeholders beyond community oral health in several countries [5, 6]. Notably, even in well-developed countries, there are instances of large disparities within different social classes [7]. Many studies have been conducted to show inequalities in oral health. Provision of dental care services is a matter of public health concern for socio-economically disadvantaged people who, as a priority group, demand more support [8].

While talking about inequality and disparity in healthcare, different determinants will be discussed. Utilization, provision, and access to services are three related different terms, and are often used interchangeably in the dental literature. Access to services has three dimensions which are financial, physical, and acceptability [9]. The utilization of medical services describes the use of services by individuals for preventive or curative care, while the provision of services deals with the process of providing services according to the various available inputs (human resources, physical capital, and consumables) in the health system [10, 11].

There are many studies around oral health disparities, but there is insufficient information around the mechanisms [12]. In two separate studies in 2018, Reda et al. systematically investigated the inequalities reported globally in the utilization of dental services, as well as the impact of sociodemographic factors on inequality [13, 14]. However, they did not consider factors affecting the provision of dental services, as a dominant element in inequality. In addition, due to their methodology for investigating the inequality in the utilization of dental services, the underlying mechanisms in inequality in dental services have not been explained by any review with a systematic approach so far.

In spite of presenting the systematic results of the aforementioned studies, since the contextual and underlying factors differ globally, it seems necessary to conduct an up-to-date study of effective social, individual, and contextual determinants of inequality in the utilization of dental services in homogeneous countries. At the same time, the concept of inequality from the perspectives of utilization and provision of dental services needs further exploration. In particular, understanding what the main determinants of inequality are from a utilization and provision of dental services perspective, while considering the socio-economic and cultural contexts.

To better investigate the aforementioned topic and to address the knowledge gaps, there is a need for a narrowed scoping review. This kind of review will enable oral health policymakers to identify the whole concept and its related determinants, as well as conducting a comprehensive map [15] to integrate the related pieces of evidence to achieve evidenced-based decision and policy making. Although at first glance, the inequalities and disparities are assumed to occur in poor and less developed contexts, this may intensify among the rich and developed countries with different levels of utilization to healthcare resources. Therefore, this review aimed to identify the most cited determinants of inequality in the utilization and provision of dental services as an outcome of the oral health system in Organization for Economic Co-operation and Development (OECD) countries to help stakeholders and policymakers with the future planning of dental services to ensure a more equitable provision of services.

## Methods

This scoping review follows the Joanna Briggs Institute (JBI) scoping reviews framework provided by Arksey and O’Malley [16, 17]. In this five-step framework, after identifying research questions and database searching, the relevant studies are selected, summarized, and synthesized. As per the framework, this study was conducted in five steps, which are described in detail below.

### Step 1—Identification of the research question

The main research question was “What are the main effective determinants of inequality in utilization and provision of dental services in OECD countries?” OECD countries define as those countries which are the member of Organization for Economic Co-operation and Development. When we are talking about the key determinants, it should be mentioned that, those are some key factors that directly or indirectly can affect the provision and utilization of dental services among the people in OECD countries that can make inequality.

According to JBI’s interpretation, a research question of a scoping review should cover the population, the concept, and the context [16]. According to the research question, the population was limited to all OECD countries. The concept included various effective determinants of inequality on utilization and provision of dental services on oral health outcomes, and the context contained the comprehensive cultural, political, and behavioral background within the population.

### Step 2—Identifying the relevant studies

Keywords for the study were selected following a preliminary review of the literature. The selected keywords, based on their relevant Medical Subject Headings (MeSH), were searched in various databases up to and including 8 Aug 2020. These databases included PubMed, International Science Indexing Web of Science (ISI WOS), Scopus, and ProQuest. The general search strategy including the main keywords is shown in Table 1. For more clarification, the search strategies syntax for each database is presented in the supplementary file. The logical operators, “OR” and “AND,” were used to increase the search sensitivity. EndNote reference manager X9 (Clarivate Analytics, Philadelphia, PA, USA) was used to manage the retrieved references.

**Table 1:** Search strategy

Search engines: PubMed, ProQuest, ISI Web of Science, Scopus	
Limits: Time: up to 8 Aug 2020, Language: English	
Strategy: #1 AND #2 AND #3	
#1 ("Dental Health Surveys" OR "Dental Care" OR "Oral Health" OR "Dental Health Services")	
#2 ("Socioeconomic Factors" OR "Hierarchy, Social" OR "Healthcare Disparities" OR "Health Status Disparities" OR "Social Determinants of Health" OR "Social Class")	
#3 ("dental services" OR "dental visits" OR "utilization" OR "provision" OR "use of services")	

### Step 3—Study selection

**Fig. 1:**
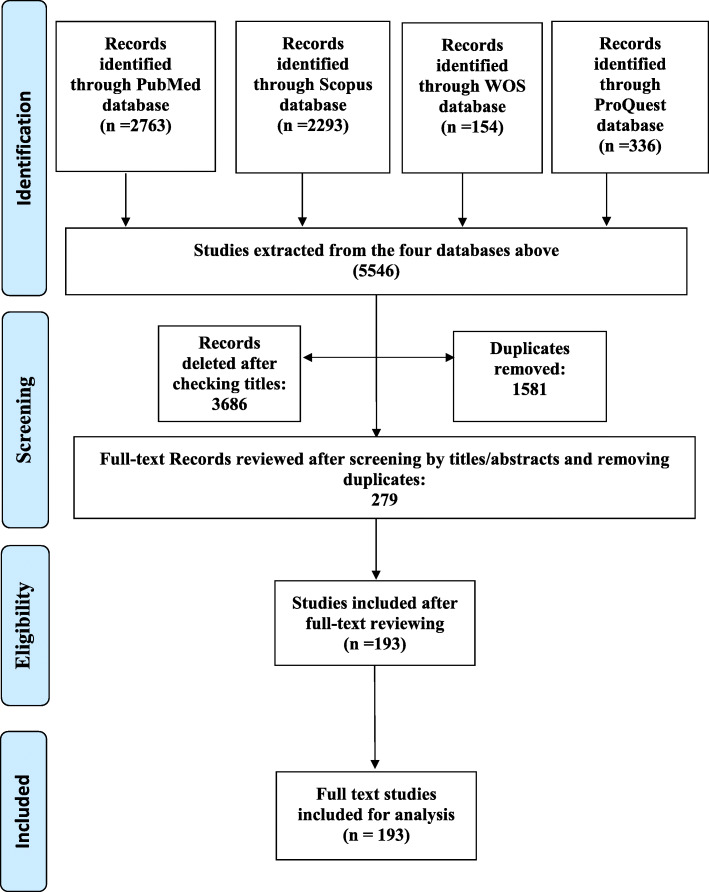
The study selection flowchart (PRISMA)

In this step, the achieved articles from the systematic search of the forth databases were reviewed first by the title and abstract and then their full texts. All of these processes were conducted by two researchers separately and independently (AG and PB). Review articles were subsequently excluded from the review process while all other types of studies such as original articles, commentaries, and letters with different methodologies (qualitative, quantitative, and mixed-method) were included. Quality appraisal of the retrieved studies is not obligatory in scoping reviews [18]. However, for assuring the eligibility of the included full-texts, another researcher (DB) screened the full-text studies, in line with the inclusion criteria and aim of the study. Figure 1 illustrates the initial search using the Preferred Reporting Items for Systematic Reviews and Meta-Analyses (PRISMA) study selection flowchart. A relevant extraction form was developed according to the review’s aim by the research team. The form was applied to extract the relevant data including the author’s name, study aim, study population, year of publication, study design, study place, and the key determinants of dental inequalities regarding the utilization and provision of the services (Table S1- supplement).

### Step 4—Charting the data

Two members of the research team (AG and PB) simultaneously and jointly charted the studies [19]. They updated the data charting form and performed the charting process in an iterative process. In this regard, the author’s name, year of publication and place of publication, study’s aim, design, main findings, and implications were extracted and charted in the data extraction form.

### Step 5—Collating, summarizing, and reporting the results

The member of the research team (PB), who was more familiar with the content-analysis, coded the studies based on a qualitative thematic approach [20]. In this regard, the initial codes were extracted from the meaningful units achieved from each article. These meaningful units were the essence of findings interpreted by the researcher (PB) and identified according to the aim of the review. Then, in a reciprocal process between the initial codes and the original extracted data and the meaningful units, the final codes emerged. Following revision, integration, and classification of the final codes, the main related themes and sub-themes were identified and subsequently tabulated. Thus, the main determinants and sub-determinants of inequality in dental services, from a utilization and provision perspective, were defined by the research team. To better understand the concept, Microsoft PowerPoint (Microsoft, Redmond, Washington, USA) was used to design a conceptual map to illustrate the concepts. In addition, viewpoints of other experts in the field were included in the final map. For assuring the rigor of qualitative data analysis, some of the criteria proposed by Guba and Lincoln were fulfilled [21]. To assure the transferability of the results that indicate the ability of transferring the findings to the similar contexts, we have tried to present a thick description of the results. Also, to reach the dependability of the results, the process of data collection and data analysis are clarified in detail to facilitate the possibility of auditing. And, finally to achieve confirmability, the researchers have tried to minimize the investigator bias via bracketing their predisposition and suppositions and differentiating them from the included data to the analysis process.

### Trend analysis

Trend analysis was added as a new step of the scoping review, as this study tries to demonstrate the trends of studies focusing on the inequality determinant. To illustrate the over-time trend of conducting studies, the publication year of the studies related to each sub-theme was specified. A Location-Time stacked bar chart was drawn using the SPSS software version 26.0 (SPSS, Chicago, Illinois, USA). To draw streamgraphs of time trends, after identifying the sub-sub-themes and articles related to each, the total number of articles in each main theme and sub-theme was determined. Then, the articles in each category were sorted based on their publication year. The streamgraphs were illustrated using PlotDB (www.plotdb.com) which is an open license website.

## Results

According to the PRISMA flowchart, in the initial search, a total of 5546 studies were identified, 1581 and 3686 studies were excluded due to duplication and mismatch of their titles respectively. Of the 279 studies that met the inclusion criteria and had their full-text reviewed, 193 full-text original studies were selected (Fig. 1).

### Thematic analysis

Thematic analysis of the included studies led to the extraction of 6 main themes, 13 sub-themes, and 53 sub-sub-themes (Table 2). All the related references are presented in Table S2- supplement. The main themes are discussed in detail below.

**Table 2:** Determinants of inequality in the utilization and provision of dental services in OECD countries

Main themes	Sub-themes	Sub-sub themes	Number of articles
**Utilization of services**			
**Individual determinants**	**Demographic determinants**	Gender	18
		Race and ethnicity	25
		Nationality/mother nationality	4
		Age	42
		Marital status	3
	**Self-rated health status**	Functional abilities	4
		Quality of life	3
		Special health needs/minorities	3
		Oral health status	10
		Disease and health status	8
**Social determinants**	**Social status**	Residential location	20
		Vulnerable groups	9
		Population density	1
		Occupation/employment	5
		Immigrant and refugees	11
	**Literacy**	Education level	32
		Health literacy	4
**Economic determinants**	**Micro-economic**	Income	58
		Wealth	7
		Poverty	11
	**Macro-economic**	Macro-economic crisis/condition	5
		Macroeconomic revenue collection	1
		Gini index	5
		GDP per capita/country revenue	2
		Economic disparities	4
**Cultural determinants**	**Macro cultural factors**	Time and technology	9
		Environmental condition	1
	**Micro cultural factors**	Oral health behavior	6
		Primary language spoken (fluency)	5
		Lifestyle	6
		Attitude	8
**Provision of services**			
**Health policy**	**Policy implementation**	Appropriate policies	7
		Target population concentration	1
		National interventions	8
	**Policy formulation**	Health basic insurance/public insurance	13
		Supplementary insurance	35
		Private insurance	5
		Cardholder status	2
**Availability of services**	**Type of available services**	Advice services/regular visiting pattern	10
		Emergency visits	7
		Service coverage	4
		Specialized services	8
		Preventive care	13
		Pharmacists consultation	1
		School dental nurses and dental hygienists	1
	**Distribution of services**	Geographic location/dentist distribution	15
		Distribution of dental schools	3
	**Management of services**	Inadequate private services	1
		Waiting time in public sector	2
		Cost of service	11
		Service satisfaction	1
		Dentists recall and follow-up	2

#### I- Individual determinants

Numerous individual factors were identified as being associated with inequality in dental service utilization. Age, as one of the main individual factors, can be the basis of different needs. This difference in need leads to the utilization of specialized treatments such as prosthetic treatments in the elderly or orthodontic treatments in children and adolescence [22, 23].

Personal conditions vary according to age. Loneliness in the elderly was also recognized as one of the factors influencing the inequality in dental service utilization [24].

Dealing with diseases or functional disabilities can lead to reduced dental service utilization [25]. One study examined the use of routine dental services in cancer patients and reported that service utilization declined even after receiving advice to increase the number of visits to the dentist [26]. Racial and ethnic factors in minorities have also been identified in many articles as determinants of dental service utilization.

#### II- Social determinants

While individual factors are more closely related to independent individual indicators, social determinants are more concerned with social factors and interactions.

Various studies claim that people living in capital areas receive more services than people living in non-capital areas, which is related to both the provision and utilization of dental services [27, 28].

Employment and its types, immigration, and education were identified as effective social determinants [29, 30]. The association between the level of health literacy and inequality in the utilization of dental services was also mentioned in some articles [31], while more attention was paid to the association between the level of academic education and the degree of utilization which was higher in people with university education and managerial jobs [32].

#### III- Economic determinants

The impact of economic conditions on both macro and micro levels was examined. At the macro-level, national economic crisis and economic differences between different parts of countries are known factors influencing inequality in utilization and provision of dental services [33, 34]. In economic crisis, the rate of utilization decreases, while after that, the situation improves [35].

At the microeconomic level, various concepts such as wealth, poverty, and income have been studied. Although there is a conceptual difference between income and wealth, in the studies that examined both, wealth was considered more related to service utilization. To clarify it, people’s wealth and assets may be higher in different cases. For instance, affordability for people who have a home without a loan or debt may differ compared to others not in a similar position [36]. However, the determinant that most studies addressed was income.

The overall analysis of the articles pointed to a direct relationship between increased income and utilization. In higher-income groups with higher socioeconomic status, the rate of utilization of dental services, as well as annual dental visits, were higher than in lower-income groups.

#### IV- Cultural determinants

Although few of the included studies investigated cultural indicators associated with the utilization of dental services, at the macro-level, various cultural factors were considered associated with the level of dental services utilization. In general, with the passage of time, improvement of environmental conditions, technological advancement, and introduction of more appropriate and wider services have been reported to be associated with an increasing dental services utilization rate [37].

At the micro-cultural level, oral health behaviors, lifestyle, and self or parents’ attitude are known to be associated factors [38]. For instance, it has been reported that people who engage in high-risk behaviors, such as smoking or alcohol consumption and who pay less attention to oral hygiene or have a negative attitude toward it, consequently, have a lower rate of utilization of dental services [25].

#### V- Health policy

Although oral health reform policies have reduced the inequality in the utilization and provision of dental services, it has still remained among the population after the implementation of the reform in most cases. Policy appropriateness for the target population and national or local level of policy implementation are reported to be associated with the utilization and provision of dental services [39].

One of the determinants that has been mentioned many times as an important enabling factor in the utilization of dental services was insurance. Being covered by insurance, whether it be primary, supplementary, private, or public, is associated with the increased utilization of dental services [40]. This determinant of course can be affected by the policy-making process from agenda setting to policy formulation and implementation.

#### VI- Availability of service

While health policies are mostly related to the provision of dental services, another determinant related to the provision of services is the availability of services. Distribution of services, distribution and density of dental schools, clinics, and dental offices, was among the sub-themes [41]. Types of available services whether public or private, long waiting time for public services, cost of services, service satisfaction, the regularity of dental visits, and follow-up sessions were other sub-themes related to the availability of services.

### Conceptual map

**Fig. 2:**
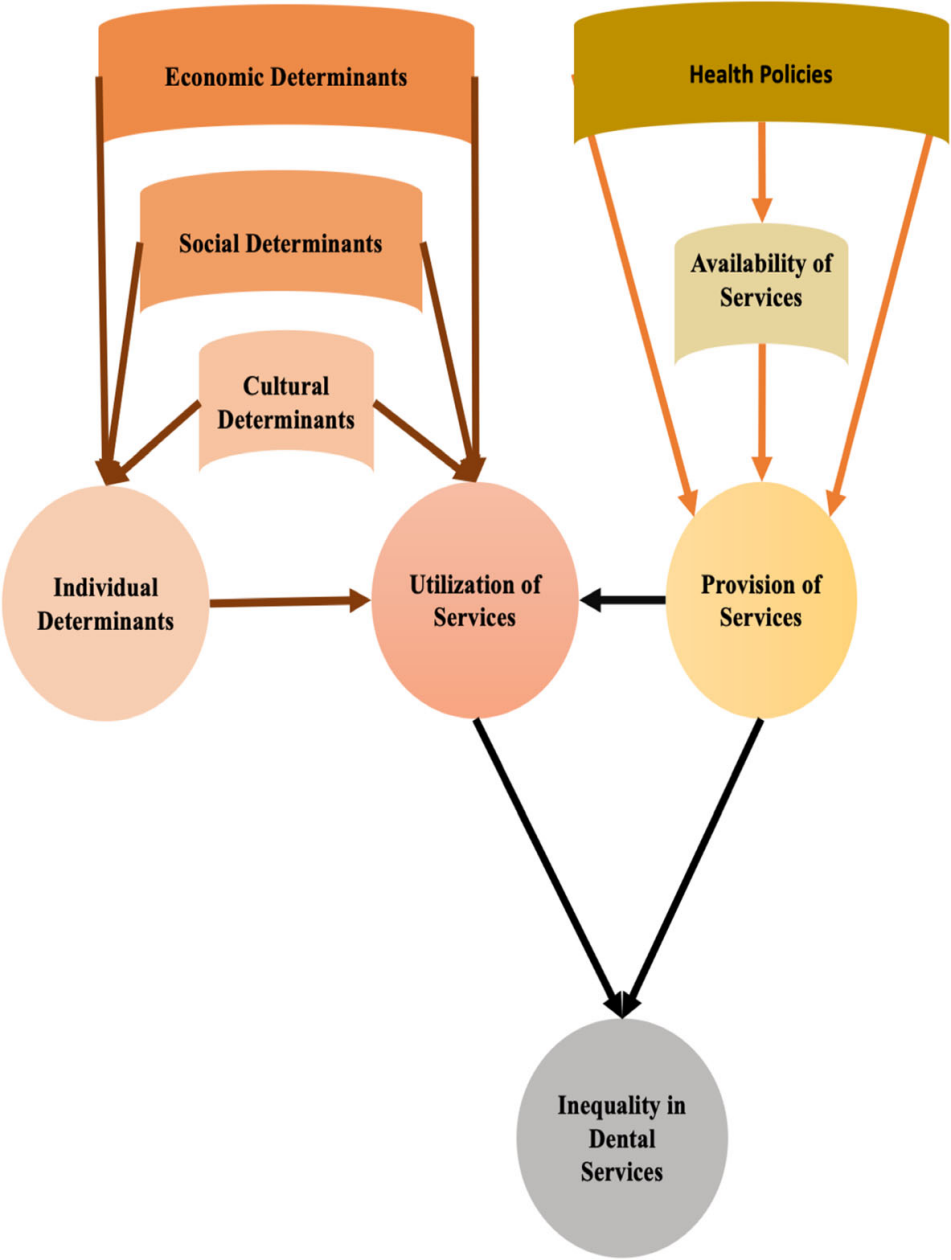
The concept map of inequality by the two dimensions of provision and utilization

Based on the main themes, the reported rational associations in the reviewed studies and consensus among the research team, the concept map of the topic was developed. It conceptualizes the determinants of inequality by the two dimensions of provision and utilization of dental services (Fig. 2). According to Fig. 2, socio-economic and cultural determinants, as external macro factors, can affect individual characteristics and utilization of services. At the same time, provision of the adequate, high quality and need-oriented services can affect service utilization. The provision of dental services is influenced by macro and micro level policies and availability of the services. Thus, inequality in the provision and utilization of dental services will be determined by their underlying determinants.

### Trend analysis

**Fig. 3:**
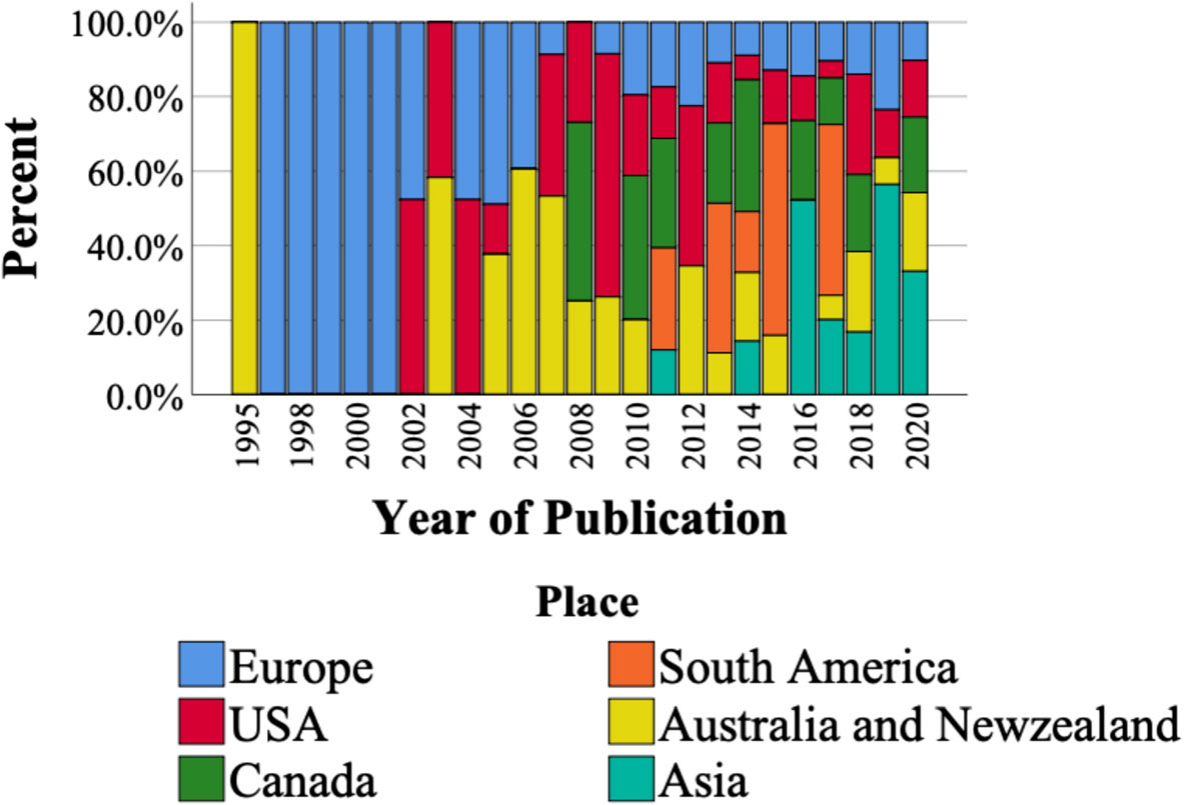
The time trend of publications based on their year and country of publication

The time trend of publications based on their proportion to the total number of published articles in the same year and stratified by country of their target population (Asia, Australia and Oceania, Canada, Europe, South America, and the USA) was obtained (Fig. 3). The lowest percentage of studies was conducted in South America (3.48%). However, the general trend shows a decrease in the share of European countries and the USA and an increase in the share of Asian and South American countries in the last decade.

**Fig. 4:**
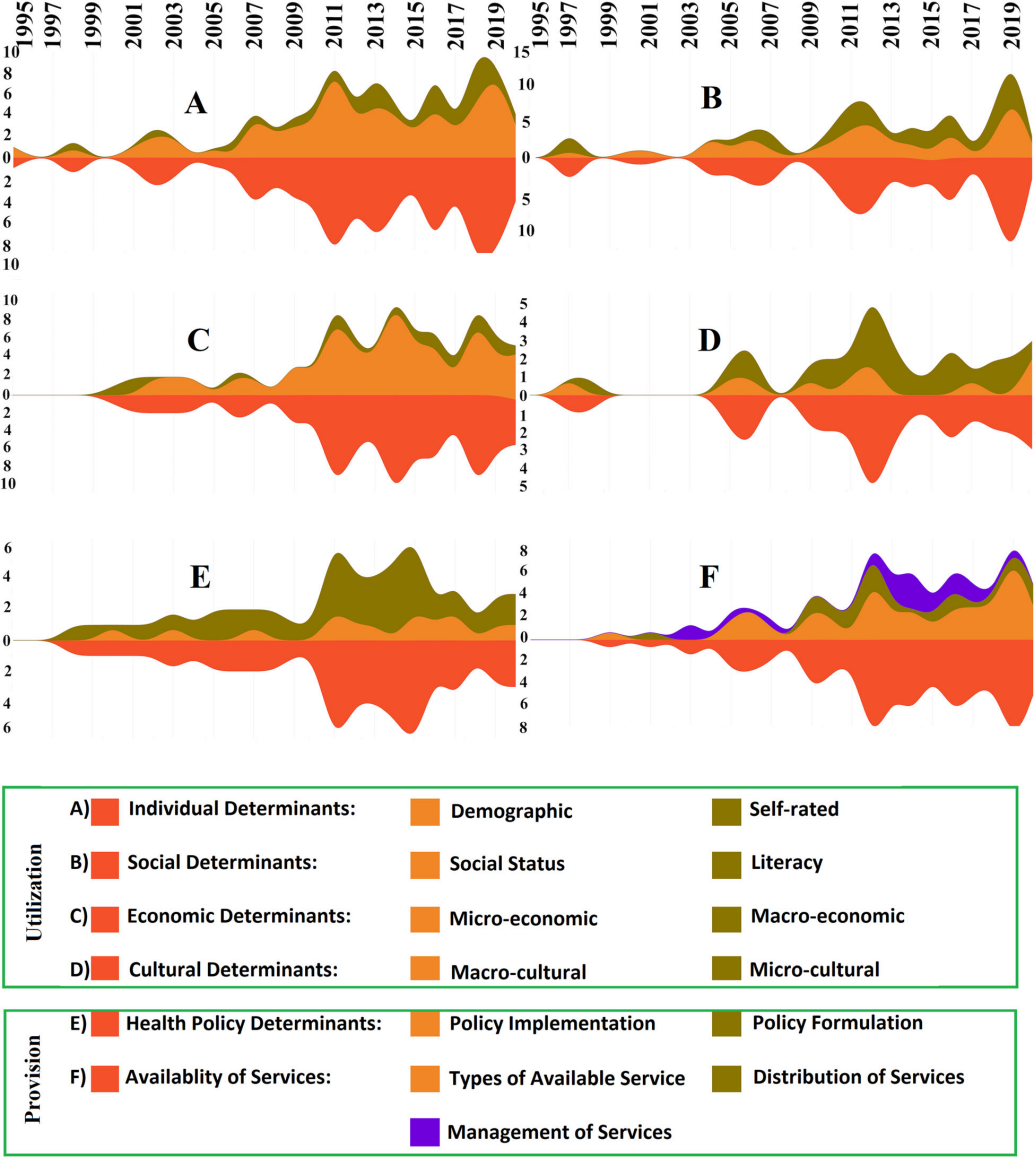
Time trend of the studies based on the main determinants of inequality

Time trend of the studies based on the main themes and the most covered sub-themes are shown in Fig. 4. In these streamgraphs, the colors blue, bright, and dark yellow are related to the sub-themes of each determinant, while orange represents the main theme, and in fact, represents the sum of the top section and total numbers of the articles related to the main theme. It illustrates that fewer studies have been conducted on social and cultural determinants, and in almost all determinants, the trend of publishing has been increasing since 2007, while studies examining health policy determinants have been decreasing since 2015.

## Discussion

Considering the present results, there is a parallel way leading to inequality in dental health. In other words, challenges in provision of appropriate and need-based dental services or lack of utilizing the services by the target population can play a role in creating inequality in dental services [42, 43].

According to the concept map, it may be argued that there is no starting point whether in the provision of services or the utilization of services which causes inequality in dental services that leads us to consider all identified determinants together to tackle the inequality in dental services. To the best of our knowledge, even lack of appropriate provision of dental services or the low level of utilization of these services among the population can lead to inequality [44]. Considering that, these results can contribute to the present knowledge that every mechanism on the area of changing or improving the policies or any action toward increasing the availability of dental services can improve the provision aspect. Notably, simultaneous attention to the macro determinants, the same as social, cultural, or economic, can enable the population to utilize different dental services. Only in the logical tradeoff among appropriate, need-oriented provision of dental services, and the adequate and effective utilization of the services can assure a more equitable oral health system.

This inference is consistent with the results of studies examining the impact of policy reforms [45–47]. Most of these studies reported a persistent inequality among the population after the reform. In comparison, Shin et al. in 2020 reported that the slope of the inequality associated with the rate of dental caries in Korean children disappeared after the introduction of the new sealant therapy policy [39].

It seems that except when all the determinants in the target community or population group are considered, inequality in dental services would not be eliminated with a reform policy in the short term [48], but if the determinants of social, cultural, economic, and availability of services are in good shape, then we can expect an improvement in equality of dental services.

At the same time, it can be considerable for health policy makers to understand whether they have to change each of the policymaking chains; agenda setting, policy formulation, policy implementation, policy evaluation; for better results and reduction of inequalities.

This logic can be seen in the study of other determinants as well. Regarding individual determinants, a 1997 study by Honkala et al. found that there was a correlation between occupation, education, and dental visits in the Finish population until 1983, but there has been no such correlation since then [33]. In another study in Australia, Singh et al. reported that there is not only a positive relationship between income and oral health outcomes but also a negative one. They attributed the contextual differences in Australia compared with other countries [49]. To put it in a nutshell, as the map shows, the way to tackle the inequality in dental services is to target all determinants at the same time.

According to the publishing trends of articles, although this trend has been consistent and increasing since 2017, it may be argued that there has not been enough research related to determinants of social, cultural, and health policies. In other words, most of the researchers’ focus on specific factors such as income caused a variety of replicated studies. Therefore, research on other aspects of inequality in dental services is pertinent.

Utilization and access to services are often used interchangeably in the dental literature. In this study, in addition to trying to differentiate between the concepts of utilization and provision of dental services, we tried to determine the determinants and sub-themes of each to design a relevant concept map to help researchers and policymakers to better address this issue. This study is one of the largest reviews in the study of inequality in dental services in terms of the number of included studies and is unique in terms of the methodology. However, due to the large number of studies reviewed, the purpose and methodology of the study, we excluded the inequality related determinants of access to dental services and should be considered separately. Therefore, the need to identify the determinants of the third edge of the “inequality triangle” in dental services (access) is still necessary.

## Conclusion

In conclusion, various factors are related to inequality in the utilization and provision of dental services. These include individual, social, cultural, and economic determinants that are related to the utilization of services. On the other hand, there are health policies and availability of services, which relate to the provision. Disparities in each of these determinants can lead to inequality in utilization and provision of dental services. In conclusion, all these aspects must be considered to reduce inequality in dental services.

## Supplementary Information

**Additional file 1: **Utilization/Provision of Dental Services Search Strategies Syntax. **Table S1.** Data extraction form of the scoping review. **Table S2.** Determinants of inequality in utilization and provision of dental services in OECD countries.

## Data Availability

While identifying/confidential patient data should not be published within the manuscript, the datasets used and/or analyzed during the current study are available from the corresponding author on reasonable request.

## Abbreviations

OECD: Organization for Economic Co-operation and Development
