# Supplementary material for: Inequalities in utilization and provision of dental services: a scoping review
Source: Syst Rev. 2021 Aug 10;10:222. doi: 10.1186/s13643-021-01779-2 (PMC8356458; doi:10.1186/s13643-021-01779-2)
Supplement: Supplementary file 1 — Additional file 1: Utilization/Provision of Dental Services Search Strategies Syntax. Table S1. Data extraction form of the scoping review. Table S2. Determinants of inequality in utilization and provision of dental services in OECD countries. [file 13643_2021_1779_MOESM1_ESM.doc]

**Utilization /Provision of Dental Services Search Strategies Syntax**

**TOTAL: 5546**

**Included 193**

**PubMed 2763**

((("Dental Health Surveys"[Mesh]) OR ( "Dental Care"[Mesh] OR ( "Oral Health"[Mesh] OR "Dental Health Services"[Mesh] )) AND ((((("Socioeconomic Factors"[Mesh]) OR "Hierarchy, Social"[Mesh]) OR ( "Healthcare Disparities"[Mesh] OR "Health Status Disparities"[Mesh] )) OR "Social Determinants of Health"[Mesh]) OR "Social Class"[Mesh])) AND (((("dental services"[Title/Abstract]) OR ("dental visits"[Title/Abstract])) OR ("utilization" [Title/Abstract])) OR ("provision" [Title/Abstract])) OR ("use of services"[Title/Abstract]))

**Scopus 2293**

TITLE-ABS-KEY("oral health") OR TITLE-ABS-KEY("Dental Health Surveys") OR TITLE-ABS-KEY("Dental Health Services") OR TITLE-ABS-KEY("dental care") AND TITLE-ABS-KEY("Socioeconomic Factors") OR TITLE-ABS-KEY("Social Hierarchy") OR TITLE-ABS-KEY(Inequalities) OR TITLE-ABS-KEY("Social Disparities") OR TITLE-ABS-KEY("Social Gradient") OR TITLE-ABS-KEY("Health Status") OR TITLE-ABS-KEY("socioeconomic disadvantage") OR TITLE-ABS-KEY("socioeconomic inequalities") OR TITLE-ABS-KEY("Social Determinants") AND TITLE-ABS-KEY("dental services") OR TITLE-ABS-KEY("dental visits") OR TITLE-ABS-KEY("utilization") OR TITLE-ABS-KEY(provision) OR TITLE-ABS-KEY("use of services")

**WOS 154**

TS= ("Dental Health Surveys" OR "Dental Care" OR "Oral Health" OR "Dental Health Services")

AND

TS= ("Socioeconomic Factors" OR "Hierarchy, Social" OR "Healthcare Disparities" OR "Health Status Disparities" OR "Social Determinants of Health" OR "Social Class")

AND

TS= ("dental services" OR "dental visits" OR "utilization" OR "provision" OR "use of services")

**ProQuest 336**

noft(("Dental Health Surveys" OR "Dental Care" OR "Oral Health" OR "Dental Health Services")) AND noft(("Socioeconomic Factors" OR "Hierarchy, Social" OR "Healthcare Disparities" OR "Health Status Disparities" OR "Social Determinants of Health" OR "Social Class") ) AND noft(("dental services" OR "dental visits" OR "utilization" OR "provision" OR "use of services"))

| **Table S1- Data extraction form of the scoping review** | | | | | | | |
| --- | --- | --- | --- | --- | --- | --- | --- |
| **No** | **Author** | **Study Title/aim** | **Study population** | **Year** | **Study Design** | **Study place** | **Key Determinants of the study**  **(in the scopes of utilization, access, provision, equality)** |
|  |  |  |  |  |  |  | - A - B - C - … |
|  |  |  |  |  |  |  |  |

**Table S2- Determinants of inequality in utilization and provision of dental services in OECD countries**

| **Main themes** | **Sub-themes** | Sub-sub themes | Count | References |
| --- | --- | --- | --- | --- |
| **Utilization of Services** | | | | |
| **Individual**  **determinants** | **Demographic determinants** | Gender | 18 | (1-18) |
|  |  | Race and ethnicity | 25 | (8, 9, 19-41) |
|  |  | Nationality /Mother nationality | 4 | (3, 4, 42, 43) |
|  |  | Age | 42 | (10, 11, 14, 15, 18, 22, 27-31, 33, 36, 38, 44-71) |
|  |  | Marital status | 3 | (3, 38, 46) |
|  | **Self-rated health status** | Functional abilities | 4 | (67, 72-74) |
|  |  | Quality of life | 3 | (75-77) |
|  |  | Special health needs/minorities | 3 | (31, 51, 78) |
|  |  | Oral health status | 10 | (8, 9, 28, 31, 47, 57, 72, 79-81) |
|  |  | Disease and health status | 8 | (5, 16, 24, 32, 37, 46, 77, 82) |
| **Social determinants** | **social status** | Residential location | 20 | (6, 14, 26, 27, 30, 36, 83-96) |
|  |  | Vulnerable groups | 9 | (8, 26, 29, 30, 37, 64, 66, 97, 98) |
|  |  | Population density | 1 | (94) |
|  |  | Occupation /employment | 5 | (12, 99-102) |
|  |  | Immigrant and refugees | 11 | (6, 8, 32, 41, 68, 71, 97, 103-106) |
|  | **Literacy** | Education level | 32 | (1, 3, 5, 10, 13, 18, 20-22, 37-39, 43, 59, 65, 72, 78, 82, 86, 99-102, 107-115) |
|  |  | Health literacy | 4 | (78, 112, 116, 117) |
| **Economic determinants** | **Micro-economic** | Income | 58 | (1, 3, 5, 9-11, 14, 17, 20-23, 33, 36-38, 43, 46, 47, 51, 52, 58, 61, 66, 73, 79, 80, 85, 86, 88, 103, 106, 107, 109, 118-141) |
|  |  | Wealth | 7 | (10, 73, 109, 124, 127, 140, 142) |
|  |  | poverty | 11 | (13, 33, 49, 54, 82, 95, 97, 120, 124, 129, 143) |
|  | **Macro-economic** | Macro-Economic crisis/condition | 5 | (100, 104, 144-146) |
|  |  | Macroeconomic revenue collection | 1 | (147) |
|  |  | Gini index | 5 | (7, 47, 79, 118, 136) |
|  |  | GDP per capita/Country revenue | 2 | (147, 148) |
|  |  | Economic disparities | 4 | (83, 84, 87, 108) |
| **Cultural determinants** | **Macro cultural factors** | Time and technology | 9 | (101, 135, 149-155) |
|  |  | Environmental condition | 1 | (84) |
|  | **Micro cultural factors** | Oral health behavior | 6 | (5, 30, 38, 57, 85, 156) |
|  |  | Primary language spoken (Fluency) | 5 | (36, 41, 68, 71, 157) |
|  |  | lifestyle | 6 | (5, 14, 57, 67, 114, 158) |
|  |  | Attitude | 8 | (32, 42, 71, 76, 77, 85, 117, 139) |
| Provision of Services | | | | |
| **Health policy** | **Policy implementation** | Appropriate policies | 7 | (63, 132-134, 143, 159, 160) |
|  |  | Target population concentration | 1 | (87) |
|  |  | National interventions | 8 | (63, 111, 141, 153, 159-163) |
|  | **Policy Formulation**  **(Insurance)** | Health basic insurance/public insurance | 13 | (11, 15, 18, 35, 38, 41, 66-69, 78, 89, 91, 106, 111, 114, 116, 119, 120, 135, 137, 138, 140, 143, 148, 155, 164-172) |
|  |  | Supplementary insurance | 35 | (126, 134, 166, 173) |
|  |  | Private insurance | 5 | (9, 18, 125, 174, 175) |
|  |  | cardholder status | 2 | (91, 176) |
| **Availability of services** | **Type of available services** | Advice services /regular visiting pattern | 10 | (15, 36, 42, 91, 139, 155, 158, 165, 168, 177) |
|  |  | Emergency visits | 7 | (68, 69, 90, 178-181) |
|  |  | Service coverage | 4 | (28, 63, 80, 182) |
|  |  | Specialized services | 8 | (7, 22, 44, 61, 84, 88, 102, 183) |
|  |  | Preventive care | 13 | (3, 11, 31, 38, 98, 99, 113, 115, 121, 164, 171, 184, 185) |
|  |  | Pharmacists Consultation | 1 | (180) |
|  |  | School dental nurses and dental hygienists | 1 | (186) |
|  | **Distribution of services** | Geographic location /dentist distribution | 15 | (11, 39, 88, 90, 93, 126, 137, 171, 172, 180, 185, 187-190) |
|  |  | Distribution of dental schools | 3 | (148, 188, 191) |
|  | **Management of services** | Inadequate private services | 1 | (182) |
|  |  | Waiting time in public sector | 2 | (105, 161) |
|  |  | Cost of service | 11 | (16, 60, 69, 91, 106, 140, 148, 157, 165, 192, 193) |
|  |  | Service Satisfaction | 1 | (179) |
|  |  | Dentists recall and follow up | 2 | (92, 130) |

**Bibliography**

1. Guiney H, Woods N, Whelton HP, Morgan K. Predictors of utilisation of dental care services in a nationally representative sample of adults. 2011.
2. Anttila J, Tolvanen M, Kankaanpaa R, Lahti S. Social gradient in intermediary determinants of oral health at school level in Finland. *Community dental health* 2018; **35**: 75-80.
3. Christensen LB, Petersen PE, Steding‐Jessen M. Consumption of dental services among adults in Denmark 1994–2003. *European journal of oral sciences* 2007; **115**: 174-179.
4. Christensen LB, Twetman S, Sundby A. Oral health in children and adolescents with different socio-cultural and socio-economic backgrounds. *Acta odontologica Scandinavica* 2010; **68**: 34-42.
5. Kengne Talla P, Gagnon MP, Dramaix M, Leveque A. Barriers to dental visits in Belgium: a secondary analysis of the 2004 National Health Interview Survey. *Journal of public health dentistry* 2013; **73**: 32-40.
6. Lee H, Seo S, Kang R, Kim Y, Hyun HK. Increasing access to oral healthcare for marriage-immigrant women in South Korea: programme design to policy recommendation. *International dental journal* 2019; **69**: 354-360.
7. Simon L, Choi SE, Ticku S, Fox K, Barrow J, Palmer N. Association of income inequality with orthodontic treatment use. *Journal of the American Dental Association* 2020; **151**: 190-196.
8. Hunter LP, Yount SM. Oral health and oral health care practices among low-income pregnant women. *Journal of midwifery & women's health* 2011; **56**: 103-109.
9. Akintobi TH, Hoffman LM, McAllister C, Goodin L, Hernandez ND, Rollins L, Miller A. Assessing the Oral Health Needs of African American Men in Low-Income, Urban Communities. *American journal of men's health* 2018; **12**: 326-337.
10. Allin S, Masseria C, Mossialos E. Equity in health care use among older people in the UK: an analysis of panel data. *Applied Economics* 2011; **43**: 2229-2239.
11. Bailit H, D'Adamo J. State case studies: improving access to dental care for the underserved. *J Public Health Dent* 2012; **72**: 221-234.
12. Caban-Martinez AJ, Lee DJ, Fleming LE, Arheart KL, Leblanc WG, Chung-Bridges K, Christ S, Pitman T. Dental care access and unmet dental care needs among U.S. workers: the National Health Interview Survey, 1997 to 2003. *J Am Dent Assoc* 2007; **138**: 227-230.
13. Carrion IV, Castaneda H, Martinez-Tyson D, Kline N. Barriers impeding access to primary oral health care among farmworker families in Central Florida. *Soc Work Health Care* 2011; **50**: 828-844.
14. Crocombe LA, Stewart JF, Brennan DS, Slade GD, Spencer AJ. Is poor access to dental care why people outside capital cities have poor oral health? *Aust Dent J* 2012; **57**: 477-485.
15. Dounis G, Ditmyer MM, McCants R, Lee Y, Mobley C. Southern Nevada assisted living residents' perception of their oral health status and access to dental care. *Gerodontology* 2012; **29**: e150-154.
16. Jones E, Shi L, Hayashi AS, Sharma R, Daly C, Ngo-Metzger Q. Access to oral health care: the role of federally qualified health centers in addressing disparities and expanding access. *Am J Public Health* 2013; **103**: 488-493.
17. Kiuchi S, Aida J, Kusama T, Yamamoto T, Hoshi M, Yamamoto T, Kondo K, Osaka K. Does public transportation reduce inequalities in access to dental care among older adults? Japan Gerontological Evaluation Study. *Community dentistry and oral epidemiology* 2020; **48**: 109-118.
18. Roberts‐Thomson K, Stewart J. Access to dental care by young South Australian adults. *Australian Dental Journal* 2003; **48**: 169-174.
19. Gilbert GH, Paul Duncan R, Shelton BJ. Social determinants of tooth loss. *Health services research* 2003; **38**: 1843-1862.
20. Gupta N, Vujicic M, Yarbrough C, Harrison B. Disparities in untreated caries among children and adults in the U.S., 2011-2014. *BMC oral health* 2018; **18**: 30.
21. Liu Y, Li Z, Walker MP. Social disparities in dentition status among American adults. *International dental journal* 2014; **64**: 52-57.
22. Moeller JF, Chen H, Manski RJ. Diversity in the use of specialized dental services by older adults in the United States. *Journal of public health dentistry* 2019; **79**: 160-174.
23. Shi L, Lebrun LA, Tsai J. Access to medical care, dental care, and prescription drugs: the roles of race/ethnicity, health insurance, and income. *Southern medical journal* 2010; **103**: 509.
24. Smith DK, Castellanos EH, Murphy BA. Financial and socio-economic factors influencing pre- and post-cancer therapy oral care. *Supportive care in cancer : official journal of the Multinational Association of Supportive Care in Cancer* 2018; **26**: 2143-2148.
25. Zhang W, Wu YY, Wu B. Racial/Ethnic Disparities in Dental Service Utilization for Foreign-Born and U.S.-Born Middle-Aged and Older Adults. *Research on aging* 2019; **41**: 845-867.
26. Martin-Kerry JM, Whelan M, Rogers J, Raichur A, Cole D, de Silva AM. Addressing disparities in oral disease in Aboriginal people in Victoria: where to focus preventive programs. *Australian Journal of Primary Health* 2019; **25**: 317-324.
27. Armfield JM. Socioeconomic inequalities in child oral health: a comparison of discrete and composite area‐based measures. *Journal of Public Health Dentistry* 2007; **67**: 119-125.
28. Dehmoobadsharifabadi A, Singhal S, Quinonez CR. Impact of public dental care spending and insurance coverage on utilization disparities among Canadian jurisdictions. *Journal of public health dentistry* 2018; **78**: 346-351.
29. Ha DH, Xiangqun J, Cecilia MG, Jason A, Do LG, Jamieson LM. Social inequality in dental caries and changes over time among Indigenous and non-Indigenous Australian children. *Australian and New Zealand journal of public health* 2016; **40**: 542-547.
30. Kilpatrick NM, Neumann A, Lucas N, Chapman J, Nicholson JM. Oral health inequalities in a national sample of Australian children aged 2-3 and 6-7 years. *Australian dental journal* 2012; **57**: 38-44.
31. Bell JF, Huebner CE, Reed SC. Oral health need and access to dental services: evidence from the National Survey of Children's Health, 2007. *Matern Child Health J* 2012; **16 Suppl 1**: S27-34.
32. DiMarco MA, Ludington SM, Menke EM. Access to and utilization of oral health care by homeless children/families. *Journal of Health Care for the Poor and Underserved* 2010; **21**: 67-81.
33. Edelstein BL, Chinn CH. Update on disparities in oral health and access to dental care for America's children. *Academic pediatrics* 2009; **9**: 415-419.
34. Flores G, Lin H. Trends in racial/ethnic disparities in medical and oral health, access to care, and use of services in US children: has anything changed over the years? *International Journal for Equity in Health* 2013; **12**: 10.
35. Flores G, Tomany-Korman SC. Racial and ethnic disparities in medical and dental health, access to care, and use of services in US children. *Pediatrics* 2008; **121**: e286-298.
36. Fulkerson ND, Haff DR, Chino M. Health care access disparities among children entering kindergarten in Nevada. *J Child Health Care* 2013; **17**: 253-263.
37. Lebrun LA, Shi L. Nativity status and access to care in Canada and the U.S.: factoring in the roles of race/ethnicity and socioeconomic status. *J Health Care Poor Underserved* 2011; **22**: 1075-1100.
38. Malecki K, Wisk LE, Walsh M, McWilliams C, Eggers S, Olson M. Oral health equity and unmet dental care needs in a population-based sample: findings from the Survey of the Health of Wisconsin. *Am J Public Health* 2015; **105 Suppl 3**: S466-474.
39. Nash DA. Improving access to oral health care for children by expanding the dental workforce to include dental therapists. *Dent Clin North Am* 2009; **53**: 469-483.
40. Okunseri C, Bajorunaite R, Matthew R, Iacopino AM. Racial and ethnic variation in the provision of dental procedures. *Journal of public health dentistry* 2007; **67**: 20-27.
41. Shelley D, Russell S, Parikh NS, Fahs M. Ethnic disparities in self-reported oral health status and access to care among older adults in NYC. *J Urban Health* 2011; **88**: 651-662.
42. Nota A, Caruso S, Cantile T, Gatto R, Ingenito A, Tecco S, Ferrazzano GF. Socioeconomic Factors and Oral Health-Related Behaviours Associated with Dental Caries in Preschool Children from Central Italy (Province of Ascoli Piceno). *BioMed research international* 2019; **2019**: 7981687.
43. Trohel G, Bertaud-Gounot V, Soler M, Chauvin P, Grimaud O. Socio-Economic Determinants of the Need for Dental Care in Adults. *PloS one* 2016; **11**: e0158842.
44. Islas-Granillo H, Borges-Yañez S, Medina-Solis C, Márquez-Rodríguez S, Lucas-Rincón S, Fernández-Barrera M, Ascencio-Villagrán A, Veras-Hernández M. Dental prosthetic treatment needs in Mexican elders: Influence of socioeconomic position. *Dental and Medical Problems* 2017; **54**: 383-387.
45. Mariño R, Cueto A, Badenier O, Acevedo R, Moya R. Oral health status and inequalities among ambulant older adults living in central Chile. *Community dental health* 2011; **28**: 143.
46. Aarabi G, Valdez R, Spinler K, Walther C, Seedorf U, Heydecke G, Konig HH, Hajek A. Determinants of Postponed Dental Visits Due to Costs: Evidence from the Survey of Health, Ageing, and Retirement in Germany. *International journal of environmental research and public health* 2019; **16**.
47. Bernabe E, Marcenes W. Income inequality and tooth loss in the United States. *Journal of dental research* 2011; **90**: 724-729.
48. Brothwell DJ, Jay M, Schönwetter DJ. Dental service utilization by independently dwelling older adults in Manitoba, Canada. *Journal of the Canadian Dental Association* 2008; **74**.
49. Costacurta M, Epis M, Docimo R. Evaluation of DMFT in paediatric patients with social vulnerability conditions. *European journal of paediatric dentistry* 2020; **21**: 70-73.
50. Kramarow EA. Dental care among adults aged 65 and over, 2017. 2019.
51. Edelstein BL. Disparities in oral health and access to care: findings of national surveys. *Ambulatory pediatrics* 2002; **2**: 141-147.
52. Grytten J, Holst D. Do young adults demand more dental services as their income increases? *Community dentistry and oral epidemiology* 2002; **30**: 463-469.
53. Hjern A, Grindefjord M, Sundberg H, Rosén M. Social inequality in oral health and use of dental care in Sweden. *Community dentistry and oral epidemiology* 2001; **29**: 167-174.
54. Jiménez-Gayosso SI, Medina-Solís CE, Lara-Carrillo E, Scougal-Vilchis RJ, de la Rosa-Santillana R, Márquez-Rodríguez S, Mendoza-Rodríguez M, de Jesús Navarrete-Hernández J. Socioeconomic inequalities in oral health service utilization any time in their lives for Mexican schoolchildren from 6 to 12 years old. *Gac Med Mex* 2015; **151**: 27-33.
55. Kim J, Choi Y, Park S, Kim JL, Lee TH, Cho KH, Park EC. Disparities in the experience and treatment of dental caries among children aged 9-18 years: the cross-sectional study of Korean National Health and Nutrition Examination Survey (2012-2013). *International journal for equity in health* 2016; **15**: 88.
56. Lambert MJ, Vanobbergen JSN, Martens LC, De Visschere LMJ. Socioeconomic inequalities in caries experience, care level and dental attendance in primary school children in Belgium: a cross-sectional survey. *BMJ open* 2017; **7**: e015042.
57. Lee YS, Kim HG, Hur JY, Yang K. Oral Health in Low-Income Older Adults in Korea. *Journal of community health nursing* 2016; **33**: 98-106.
58. Listl S. Income-related inequalities in dental service utilization by Europeans aged 50+. *Journal of dental research* 2011; **90**: 717-723.
59. Listl S. Inequalities in dental attendance throughout the life-course. *Journal of dental research* 2012; **91**: 91S-97S.
60. Manski RJ, Hyde JS, Chen H, Moeller JF. Differences Among Older Adults in the Types of Dental Services Used in the United States. *Inquiry : a journal of medical care organization, provision and financing* 2016; **53**.
61. Matsuyama Y, Aida J, Takeuchi K, Tsakos G, Watt RG, Kondo K, Osaka K. Inequalities of dental prosthesis use under universal healthcare insurance. *Community dentistry and oral epidemiology* 2014; **42**: 122-128.
62. Roberts‐Thomson K, Brennan DS, Spencer AJ. Social inequality in the use and comprehensiveness of dental services. *Australian journal of public health* 1995; **19**: 80-85.
63. Shin BM, Jung SH, Kim MH, Ryu JI. Did the extended coverage policy contribute to alleviating socioeconomic inequality in untreated dental caries of both children and adolescents in South Korea? *BMC oral health* 2020; **20**: 124.
64. Verlinden DA, Reijneveld SA, Lanting CI, van Wouwe JP, Schuller AA. Socio-economic inequality in oral health in childhood to young adulthood, despite full dental coverage. *European journal of oral sciences* 2019; **127**: 248-253.
65. Vikum E, Krokstad S, Holst D, Westin S. Socioeconomic inequalities in dental services utilisation in a Norwegian county: the third Nord-Trondelag Health Survey. *Scandinavian journal of public health* 2012; **40**: 648-655.
66. Amin MS, Perez A, Nyachhyon P. Barriers to utilization of dental services for children among low-income families in Alberta. *J Can Dent Assoc* 2014; **80**: e51.
67. Österberg T, Lundgren M, Emilson C-G, Sundh V, Birkhed D, Steen B. Utilization of dental services in relation to socioeconomic and health factors in the middle-aged and elderly Swedish population. *Acta Odontologica Scandinavica* 1998; **56**: 41-47.
68. Guendelman S, Angulo V, Wier M, Oman D. Overcoming the odds: access to care for immigrant children in working poor families in California. *Matern Child Health J* 2005; **9**: 351-362.
69. Jones K. An evaluation of the discriminant and predictive validity of relative social disadvantage as screening criteria for priority access to public general dental care, in Australia. *BMC Health Services Research* 2014; **14**: 1-9.
70. Wanyonyi KL, Radford DR, Gallagher JE. The relationship between access to and use of dental services following expansion of a primary care service to embrace dental team training. *Public Health* 2013; **127**: 1028-1033.
71. Council on Access P, Interprofessional R, Robinson LA. Private sector response to improving oral health care access. *Dent Clin North Am* 2009; **53**: 523-535.
72. Listl S. Cost‐related dental non‐attendance in older adulthood: evidence from eleven European countries and Israel. *Gerodontology* 2016; **33**: 253-259.
73. Murakami K, Hashimoto H. Wealth-related versus income-related inequalities in dental care use under universal public coverage: a panel data analysis of the Japanese Study of Aging and Retirement. *BMC public health* 2016; **16**: 24.
74. Shin HS. Social gradients in oral health status in Korea population. *Archives of oral biology* 2018; **95**: 89-94.
75. Vásquez F, Paraje G, Estay M. Income-related inequality in health and health care utilization in Chile, 2000-2009. *Revista Panamericana de Salud Pública* 2013; **33**: 98-106.
76. Valdez R, Aarabi G, Spinler K, Walther C, Seedorf U, Heydecke G, Buczak-Stec E, Konig HH, Hajek A. Association between Subjective Well-Being and Frequent Dental Visits in the German Ageing Survey. *International journal of environmental research and public health* 2020; **17**.
77. Rocha-Buelvas A, Hidalgo-Troya A, Hidalgo-Eraso Á. Barriers of access to oral health care among university students in southern Colombia, 2011. A multivariate analysis. *Revista de la Facultad de Medicina* 2014; **62**: 521-528.
78. Sohn W, Ismail A, Amaya A, Lepkowski J. Determinants of dental care visits among low-income African-American children. *Journal of the American Dental Association* 2007; **138**: 309-318; quiz 395-396, 398.
79. Moeller J, Quinonez C. The Association Between Income Inequality and Oral Health in Canada: A Cross-Sectional Study. *International journal of health services : planning, administration, evaluation* 2016; **46**: 790-809.
80. Gundgaard J. Income-related inequality in utilization of health services in Denmark: evidence from Funen County. *Scandinavian journal of public health* 2006; **34**: 462-471.
81. Crete P, Boyd LD, Fitzgerald JK, LaSpina LM. Access to preventive oral health services for homebound populations: A pilot program. *American Dental Hygienists' Association* 2018; **92**: 24-32.
82. Stevens GD, Seid M, Tsai K-Y, West-Wright C, Cousineau MR. Improvements in access to care for vulnerable children in California between 2001 and 2005. *Public Health Reports* 2009; **124**: 682-691.
83. Salomon-Ibarra CC, Ravaghi V, Hill K, Jones CM, Landes DP, Morris AJ. Low rates of dental attendance by the age of one and inequality between local government administrative areas in England. *Community dental health* 2019; **36**: 22-26.
84. Morris E, Landes D. The equity of access to orthodontic dental care for children in the North East of England. *Public health* 2006; **120**: 359-363.
85. Piotrowska DE, Pedzinski B, Jankowska D, Huzarska D, Charkiewicz AE, Szpak AS. Socio-economic inequalities in the use of dental care in urban and rural areas in Poland. *Annals of agricultural and environmental medicine : AAEM* 2018; **25**: 512-516.
86. Piotrowska DE, Jankowska D, Huzarska D, Szpak AS, Pedzinski B. Socioeconomic inequalities in use and non-use of dental services in Poland. *International journal of public health* 2020.
87. Tickle M, Moulding G, Milsom K, Blinkhorn A. Socioeconomic and geographical influences on primary dental care preferences in a population of young children. *British dental journal* 2000; **188**: 559-562.
88. Christensen LB, Rosing K, Lempert SM, Hede B. Patterns of dental services and factors that influence dental services among 64-65-year-old regular users of dental care in Denmark. *Gerodontology* 2016; **33**: 79-88.
89. Byck GR, Walton SM, Cooksey JA. Access to dental care services for Medicaid children: variations by urban/rural categories in Illinois. *The Journal of Rural Health* 2002; **18**: 512-520.
90. Dyer T, Skinner J, Canning D, Green J. A health equity methodology for auditing oral health and NHS General Dental Services in Sheffield, England. *Community Dent Health* 2010; **27**: 68-73.
91. Ellershaw A. Oral health and access to dental care in Australia—Comparisons by cardholder status and geographic region. *Australian dental journal* 2005; **50**: 282-285.
92. Friedman JW, Mathu-Muju KR. Dental therapists: improving access to oral health care for underserved children. *American journal of public health* 2014; **104**: 1005-1009.
93. Jean G, Kruger E, Tennant M. Universal access to oral health care for Australian children: comparison of travel times to public dental services at consecutive census dates as an indicator of progressive realisation. *Aust J Prim Health* 2020; **26**: 109-116.
94. McKernan SC, Kuthy RA, Momany ET, McQuistan MR, Hanley PF, Jones MP, Damiano PC. Geographic accessibility and utilization of orthodontic services among Medicaid children and adolescents. *J Public Health Dent* 2013; **73**: 56-64.
95. Pinilla J, Gonzalez B. Equity in children's utilization of dental services: effect of a children's dental care programme. *Community dental health* 2006; **23**: 152.
96. Willie-Stephens J, Kruger E, Tennant M. Public and private dental services in NSW: a geographic information system analysis of access to care for 7 million Australians. *New South Wales Public Health Bulletin* 2014; **24**: 164-170.
97. Ferrazzano GF, Cantile T, Sangianantoni G, Ingenito A, Rengo S, Alcidi B, Spagnuolo G. Oral health status and Unmet Restorative Treatment Needs (UTN) in disadvantaged migrant and not migrant children in Italy. *European journal of paediatric dentistry* 2019; **20**: 10-14.
98. Batliner TS. American Indian and Alaska Native Access to Oral Health Care: A Potential Solution. *Journal of Health Care for the Poor and Underserved* 2016; **27**: 1-10.
99. Cheema J, Sabbah W. Inequalities in preventive and restorative dental services in England, Wales and Northern Ireland. *British dental journal* 2016; **221**: 235-239.
100. Leinsalu M, Reile R, Vals K, Petkeviciene J, Tekkel M, Stickley A. Macroeconomic changes and trends in dental care utilization in Estonia and Lithuania in 2004-2012: a repeated cross-sectional study. *BMC oral health* 2018; **18**: 199.
101. Honkala E, Kuusela S, Rimpelä A, Rimpelä M, Jokela J. Dental services utilization between 1977 and 1995 by Finnish adolescents of different socioeconomic levels. *Community dentistry and oral epidemiology* 1997; **25**: 385-390.
102. Telford C, Murray L, Donaldson M, O'Neill C. An analysis examining socio-economic variations in the provision of NHS general dental practitioner care under a fee for service contract among adolescents: Northern Ireland Longitudinal Study. *Community dentistry and oral epidemiology* 2012; **40**: 70-79.
103. Rhee Kim YO, Telleen S. Predictors of the utilization of oral health services by children of low-income families in the United States: beliefs, cost, or provider? *Journal of Korean Academy of Nursing* 2004; **34**: 1460-1467.
104. Rodriguez-Alvarez E, Lanborena N, Borrell LN. Place of Birth Inequalities in Dental Care Use before and after the Economic Crisis in Spain. *International journal of environmental research and public health* 2019; **16**.
105. Davidson N, Skull S, Calache H, Chesters D, Chalmers J. Equitable access to dental care for an at‐risk group: a review of services for Australian refugees. *Australian and New Zealand journal of public health* 2007; **31**: 73-80.
106. Howard JR, Ramirez J, Li Y, Gany F. Dental care access for low-income and immigrant cancer patients in New York City. *J Community Health* 2015; **40**: 110-115.
107. Abbas H, Aida J, Saito M, Tsakos G, Watt RG, Koyama S, Kondo K, Osaka K. Income or education, which has a stronger association with dental implant use in elderly people in Japan? *International dental journal* 2019; **69**: 454-462.
108. Carta G, Cagetti M, Sale S, Congiu G, Strohmenger L, Oleari F, Bossù M, Lingström P, Campus G. Oral health inequalities in Italian schoolchildren-a cross-sectional evaluation. *Community Dent Health* 2014; **31**: 123-128.
109. Shen J, Listl S. Investigating social inequalities in older adults' dentition and the role of dental service use in 14 European countries. *The European journal of health economics : HEPAC : health economics in prevention and care* 2018; **19**: 45-57.
110. Tapias‐Ledesma MA, Jimenez R, Garrido PC, Miguel AGd. Influence of sociodemographic variables on dental service utilization and oral health among the children included in the year 2001 Spanish National Health Survey. *Journal of public health dentistry* 2005; **65**: 215-220.
111. Cornejo-Ovalle M, Paraje G, Vasquez-Lavin F, Perez G, Palencia L, Borrell C. Changes in socioeconomic inequalities in the use of dental care following major healthcare reform in Chile, 2004-2009. *International journal of environmental research and public health* 2015; **12**: 2823-2836.
112. Gupta A, Feldman S, Perkins RB, Stokes A, Sankar V, Villa A. Predictors of dental care use, unmet dental care need, and barriers to unmet need among women: results from NHANES, 2011 to 2016. *Journal of public health dentistry* 2019; **79**: 324-333.
113. Kino S, Bernabe E, Sabbah W. Social Inequalities in Use of Preventive Dental and Medical Services among Adults in European Countries. *International journal of environmental research and public health* 2019; **16**.
114. Ellershaw A. Oral health and access to dental care in Australia-comparisons by level of education. 2006.
115. Everaars B, Jerkovic-Cosic K, van der Putten GJ, Pretty IA, Brocklehurst P. Needs in Service Provision for Oral Health Care in Older People: A Comparison Between Greater Manchester (United Kingdom) and Utrecht (the Netherlands). *Int J Health Serv* 2018; **48**: 663-684.
116. Jones K, Parker E, Jamieson L. Access, literacy and behavioural correlates of poor self-rated oral health amongst an indigenous south Australian population. *Community Dent Health* 2014; **31**: 167-171.
117. Slack-Smith L, Lange A, Paley G, O'Grady M, French D, Short L. Oral health and access to dental care: a qualitative investigation among older people in the community. *Gerodontology* 2010; **27**: 104-113.
118. Aida J, Kondo K, Kondo N, Watt RG, Sheiham A, Tsakos G. Income inequality, social capital and self-rated health and dental status in older Japanese. *Social science & medicine* 2011; **73**: 1561-1568.
119. Anikeeva O, Brennan DS, Teusner DN. Household income modifies the association of insurance and dental visiting. *BMC health services research* 2013; **13**: 432.
120. Duncan L, Bonner A. Effects of income and dental insurance coverage on need for dental care in Canada. *J Can Dent Assoc* 2014; **80**: e6.
121. Grignon M, Hurley J, Wang L, Allin S. Inequity in a market-based health system: Evidence from Canada's dental sector. *Health policy* 2010; **98**: 81-90.
122. Grytten J, Holst D, Skau I. Demand for and utilization of dental services according to household income in the adult population in Norway. *Community dentistry and oral epidemiology* 2012; **40**: 297-305.
123. Guessous I, Theler J-M, Izart CD, Stringhini S, Bodenmann P, Gaspoz J-M, Wolff H. Forgoing dental care for economic reasons in Switzerland: a six-year cross-sectional population-based study. *BMC Oral Health* 2014; **14**: 121.
124. Kailembo A, Quinonez C, Lopez Mitnik GV, Weintraub JA, Stewart Williams J, Preet R, Iafolla T, Dye BA. Income and wealth as correlates of socioeconomic disparity in dentist visits among adults aged 20 years and over in the United States, 2011-2014. *BMC oral health* 2018; **18**: 147.
125. Locker D, Maggirias J, Quinonez C. Income, dental insurance coverage, and financial barriers to dental care among Canadian adults. *Journal of public health dentistry* 2011; **71**: 327-334.
126. Lupi-Pegurier L, Clerc-Urmes I, Abu-Zaineh M, Paraponaris A, Ventelou B. Density of dental practitioners and access to dental care for the elderly: a multilevel analysis with a view on socio-economic inequality. *Health policy* 2011; **103**: 160-167.
127. Manski RJ, Moeller JF, Chen H, St Clair PA, Schimmel J, Pepper JV. Wealth effect and dental care utilization in the United States. *Journal of public health dentistry* 2012; **72**: 179-189.
128. Murakami K, Aida J, Ohkubo T, Hashimoto H. Income-related inequalities in preventive and curative dental care use among working-age Japanese adults in urban areas: a cross-sectional study. *BMC Oral Health* 2014; **14**: 117.
129. Nasseh K, Vujicic M. The effect of growing income disparities on U.S. adults' dental care utilization. *Journal of the American Dental Association* 2014; **145**: 435-442.
130. Nguyen L, Häkkinen U. Income-related inequality in the use of dental services in Finland. *Applied health economics and health policy* 2004; **3**: 251-262.
131. Nishide A, Fujita M, Sato Y, Nagashima K, Takahashi S, Hata A. Income-Related Inequalities in Access to Dental Care Services in Japan. *International journal of environmental research and public health* 2017; **14**.
132. Raittio E, Aromaa A, Kiiskinen U, Helminen S, Suominen AL. Income-related inequality in perceived oral health among adult Finns before and after a major dental subsidization reform. *Acta odontologica Scandinavica* 2016; **74**: 348-354.
133. Raittio E, Kiiskinen U, Helminen S, Aromaa A, Suominen AL. Income-related inequality and inequity in the use of dental services in Finland after a major subsidization reform. *Community dentistry and oral epidemiology* 2015; **43**: 240-254.
134. Ramraj C, Sadeghi L, Lawrence HP, Dempster L, Quinonez C. Is accessing dental care becoming more difficult? Evidence from Canada's middle-income population. *PloS one* 2013; **8**: e57377.
135. Ravaghi V, Farmer J, Quinonez C. Persistent but narrowing oral health care inequalities in Canada from 2001 through 2016. *Journal of the American Dental Association* 2020; **151**: 349-357 e341.
136. Singh A, Harford J, Antunes JLF, Peres MA. Area-level income inequality and oral health among Australian adults-A population-based multilevel study. *PloS one* 2018; **13**: e0191438.
137. Biordi DL, Heitzer M, Mundy E, DiMarco M, Thacker S, Taylor E, Huff M, Marino D, Fitzgerald K. Improving access and provision of preventive oral health care for very young, poor, and low-income children through a new interdisciplinary partnership. *Am J Public Health* 2015; **105 Suppl 2**: e23-29.
138. Felland LE, Felt-Lisk S, McHugh M. Health care access for low-income people: significant safety net gaps remain. *Issue Brief Cent Stud Health Syst Change* 2004; **84**: 1-4.
139. Telleen S, Rhee Kim YO, Chavez N, Barrett RE, Hall W, Gajendra S. Access to oral health services for urban low-income Latino children: social ecological influences. *J Public Health Dent* 2012; **72**: 8-18.
140. Thompson B, Cooney P, Lawrence H, Ravaghi V, Quinonez C. Cost as a barrier to accessing dental care: findings from a Canadian population-based study. *Journal of public health dentistry* 2014; **74**: 210-218.
141. Wang H, Norton EC, Rozier RG. Effects of the State Children's Health Insurance Program on access to dental care and use of dental services. *Health Serv Res* 2007; **42**: 1544-1563.
142. Allin S, Masseria C, Mossialos E. Measuring socioeconomic differences in use of health care services by wealth versus by income. *American journal of public health* 2009; **99**: 1849-1855.
143. Newacheck PW, Hung YY, Jane Park M, Brindis CD, Irwin Jr CE. Disparities in adolescent health and health care: does socioeconomic status matter? *Health services research* 2003; **38**: 1235-1252.
144. Calzon Fernandez S, Fernandez Ajuria A, Martin JJ, Murphy MJ. The impact of the economic crisis on unmet dental care needs in Spain. *J Epidemiol Community Health* 2015; **69**: 880-885.
145. Elstad JI. Dental care coverage and income-related inequalities in foregone dental care in Europe during the great recession. *Community dentistry and oral epidemiology* 2017; **45**: 296-302.
146. Romaire MA, Bell JF, Grossman DC. Health care use and expenditures associated with access to the medical home for children and youth. *Medical care* 2012: 262-269.
147. Grytten J, Lund E, Rongen G. Equity in access to public dental services: the experience from Norway. *Acta Odontologica Scandinavica* 2001; **59**: 372-378.
148. Pegon-Machat E, Faulks D, Eaton KA, Widstrom E, Hugues P, Tubert-Jeannin S. The healthcare system and the provision of oral healthcare in EU Member States: France. *Br Dent J* 2016; **220**: 197-203.
149. Elani HW, Harper S, Allison PJ, Bedos C, Kaufman JS. Socio-economic inequalities and oral health in Canada and the United States. *Journal of dental research* 2012; **91**: 865-870.
150. FitzGerald EM, Cunich M, Clarke PM. Changes in inequalities of access to dental care in Australia 1977–2005. *Australian Economic Review* 2011; **44**: 153-166.
151. Habicht J, Kunst AE. Social inequalities in health care services utilisation after eight years of health care reforms: a cross-sectional study of Estonia, 1999. *Social science & medicine* 2005; **60**: 777-787.
152. Sengupta K, Christensen LB, Mortensen LH, Skovgaard LT, Andersen I. Trends in socioeconomic inequalities in oral health among 15-year-old Danish adolescents during 1995-2013: A nationwide, register-based, repeated cross-sectional study. *Community dentistry and oral epidemiology* 2017; **45**: 458-468.
153. Currie R, Pretty I, Tickle M, Maupomé G. Letter from America: UK and US state-funded dental provision. *Community dental health* 2012; **29**: 315.
154. Fricton J, Chen H. Using teledentistry to improve access to dental care for the underserved. *Dent Clin North Am* 2009; **53**: 537-548.
155. Smith BJ, Helgeson M, Prosa B, Finlayson TL, Orozco M, Asgari P, Pierce I, Norman G, Aronoff-Spencer E. Longitudinal analysis of cost and dental utilization patterns for older adults in outpatient and long-term care settings in Minnesota. *PloS one* 2020; **15**: e0232898.
156. Hakeem FF, Sabbah W. Is there socioeconomic inequality in periodontal disease among adults with optimal behaviours. *Acta odontologica Scandinavica* 2019; **77**: 400-407.
157. Gross–Panico ML, Freeman WK. Identifying barriers to receiving preventive dental services: expanding access to preventive dental hygiene services through affiliated practice. *American Dental Hygienists' Association* 2012; **86**: 306-314.
158. Wamala S, Merlo J, Bostrom G. Inequity in access to dental care services explains current socioeconomic disparities in oral health: the Swedish National Surveys of Public Health 2004-2005. *Journal of epidemiology and community health* 2006; **60**: 1027-1033.
159. Celeste RK, Nadanovsky P, Fritzell J. Trends in socioeconomic disparities in the utilization of dental care in Brazil and Sweden. *Scandinavian journal of public health* 2011; **39**: 640-648.
160. Leck V, Randall GE. The rise and fall of dental therapy in Canada: a policy analysis and assessment of equity of access to oral health care for Inuit and First Nations communities. *Int J Equity Health* 2017; **16**: 131.
161. Jang Y-E, Kim C-B, Kim N-H. Utilization of preventive dental services before and after health insurance covered dental scaling in Korea: 2009 to 2014 Community Health Survey. *Asia Pacific Journal of Public Health* 2017; **29**: 70-80.
162. Kim ES, Kim BI, Jung HI. Does the national dental scaling policy reduce inequalities in dental scaling usage? A population-based quasi-experimental study. *BMC oral health* 2019; **19**: 185.
163. Petersen PE, Kwan S. Equity, social determinants and public health programmes--the case of oral health. *Community Dent Oral Epidemiol* 2011; **39**: 481-487.
164. Abdus S, Decker SL. Association between Medicaid adult nonemergency dental benefits and dental services use and expenditures. *Journal of the American Dental Association* 2019; **150**: 24-33.
165. Lutfiyya MN, Gross AJ, Soffe B, Lipsky MS. Dental care utilization: examining the associations between health services deficits and not having a dental visit in past 12 months. *BMC public health* 2019; **19**: 265.
166. Srivastava P, Chen G, Harris A. Oral Health, Dental Insurance and Dental Service use in Australia. *Health economics* 2017; **26**: 35-53.
167. Teusner DN, Brennan DS, Spencer AJ. Associations between level of private dental insurance cover and favourable dental visiting by household income. *Australian dental journal* 2015; **60**: 479-489.
168. Tickle M, Williams M, Jenner T, Blinkhorn A. The effects of socioeconomic status and dental attendance on dental caries' experience, and treatment patterns in 5-year-old children. *British dental journal* 1999; **186**: 135-137.
169. Greenberg BJ, Kumar JV, Stevenson H. Dental case management: increasing access to oral health care for families and children with low incomes. *J Am Dent Assoc* 2008; **139**: 1114-1121.
170. Kotagal M, Carle AC, Kessler LG, Flum DR. Limited impact on health and access to care for 19- to 25-year-olds following the Patient Protection and Affordable Care Act. *JAMA Pediatr* 2014; **168**: 1023-1029.
171. Lin M, Sappenfield W, Hernandez L, Clark C, Liu J, Collins J, Carle AC. Child- and state-level characteristics associated with preventive dental care access among U.S. children 5-17 years of age. *Matern Child Health J* 2012; **16 Suppl 2**: 320-329.
172. Schrimshaw EW, Siegel K, Wolfson NH, Mitchell DA, Kunzel C. Insurance-related barriers to accessing dental care among African American adults with oral health symptoms in Harlem, New York City. *Am J Public Health* 2011; **101**: 1420-1428.
173. Garrido-Cumbrera M, Borrell C, Palencia L, Espelt A, Rodriguez-Sanz M, Pasarin MI, Kunst A. Social class inequalities in the utilization of health care and preventive services in Spain, a country with a national health system. *International journal of health services : planning, administration, evaluation* 2010; **40**: 525-542.
174. Ku L. Medical and dental care utilization and expenditures under Medicaid and private health insurance. *Medical care research and review* 2009; **66**: 456-471.
175. Bisgaier J, Cutts DB, Edelstein BL, Rhodes KV. Disparities in child access to emergency care for acute oral injury. *Pediatrics* 2011; **127**: e1428-1435.
176. Schwarz E. Access to oral health care–an Australian perspective. *Community Dentistry and Oral Epidemiology* 2006; **34**: 225-231.
177. Ahmadi O, Machuca C, Sabbah W. Socioeconomic inequality in the provision of health advice in dental setting in England, Wales and Northern Ireland. *Patient education and counseling* 2019; **102**: 2068-2072.
178. Kim PC, Zhou W, McCoy SJ, McDonough IK, Burston B, Ditmyer M, Shen JJ. Factors Associated with Preventable Emergency Department Visits for Nontraumatic Dental Conditions in the U.S. *International journal of environmental research and public health* 2019; **16**.
179. Al-Haboubi M, Klass C, Jones K, Bernabe E, Gallagher JE. Inequalities in the use of dental services among adults in inner South East London. *European journal of oral sciences* 2013; **121**: 176-181.
180. Cohen LA. The role of non-dental health professionals in providing access to dental care for low-income and minority patients. *Dent Clin North Am* 2009; **53**: 451-468.
181. Truong A, Higgs P, Cogger S, Dietze P. Further research required to determine unique factors associated with dental care access among deprived populations. *Public Health* 2014; **128**: 1131-1133.
182. Gallego F, Larroulet C, Palomer L, Repetto A, Verdugo D. Socioeconomic inequalities in self-perceived oral health among adults in Chile. *International journal for equity in health* 2017; **16**: 23.
183. Ravaghi V, Al-Hammadi Z, Landes D, Hill K, Morris AJ. Inequalities in orthodontic outcomes in England: treatment utilisation, subjective and normative need. *Community dental health* 2019; **36**: 198-202.
184. Shaban R, Kassim S, Sabbah W. Socioeconomic inequality in the provision of specific preventive dental interventions among children in the UK: Children's Dental Health Survey 2003. *British dental journal* 2017; **222**: 865-869.
185. Yoon H, Jang Y, Choi K, Kim H. Preventive Dental Care Utilization in Asian Americans in Austin, Texas: Does Neighborhood Matter? *International journal of environmental research and public health* 2018; **15**.
186. Nash DA. Adding dental therapists to the health care team to improve access to oral health care for children. *Academic pediatrics* 2009; **9**: 446-451.
187. Jean G, Kruger E, Tennant M. The distribution of allied dental practitioners in australia: socio-economics and rurality as a driver of better health service accessibility. *Australian dental journal* 2019; **64**: 153-160.
188. Moles D, Frost C, Grundy C. Inequalities in availability of National Health Service general dental practitioners in England and Wales. *British dental journal* 2001; **190**: 548-553.
189. Jean G, Kruger E, Tennant M. The distribution of dentists in Australia Socio-economic profile as an indicator of access to services. *Community Dent Health* 2020; **37**: 5-11.
190. Landes DP, Holmes RD. Dental practice populations: the effect of distance on the most socially deprived communities accessing dental care in the North East of England. *Public Health* 2012; **126**: 424-426.
191. Wanchek T, Rephann T. Effects of a proposed rural dental school on regional dental workforce and access to care. 2013.
192. Molarius A, Engström S, Flink H, Simonsson B, Tegelberg Å. Socioeconomic differences in self-rated oral health and dental care utilisation after the dental care reform in 2008 in Sweden. *BMC oral health* 2014; **14**: 134.
193. Tubert‐Jeannin S, Riordan PJ, Morel‐Papernot A, Moulin R. Dental status and oral health quality of life in economically disadvantaged French adults. *Special Care in Dentistry* 2004; **24**: 264-269.
